# The Dynamic Effect of Non-CYP3A4-Metabolized and CYP3A4-Metabolized Statins on Clopidogrel Resistance in Patients With Cerebral Infarction

**DOI:** 10.3389/fphar.2021.738562

**Published:** 2021-10-06

**Authors:** Hong Ting Shi, Yong Yuan Chen, Xiao Ying Li, Jian Hua Luo, Guang Hong Zhong, Jia Jia Hu, Min Zhang, Bo Rong Zhou

**Affiliations:** ^1^ Department of Neurology, The Second Affiliated Hospital of Guangzhou Medical University, Guangzhou, China; ^2^ Department of Neurology, The Fifth Affiliated Hospital of Guangzhou Medical University, Guangzhou, China; ^3^ Department of Neurology, The Shunde Affiliated Hospital of Jinan University, Shunde, China; ^4^ Department of Neurology, Yangchun People Hospital, Yangchun, China; ^5^ Department of Neurology, Heyuan People Hospital, Heyuan, China; ^6^ Department of Neurology, The Third Affiliated Hospital of Guangzhou Medical University, Guangzhou, China; ^7^ Department of Epidemiology and Health Statistics, Guangdong Pharmaceutical University, Guangzhou, China

**Keywords:** dynamic changes, CYP3A4-metabolized, clopidogrel resistance, statins, clopidogrel thiol metabolite (H4), cerebral infarction

## Abstract

**Objective:** To explore the treatment effect of statins used together with clopidogrel on cerebral infarction (CI).

**Methods:** One hundred and thirty non-clopidogrel resistant patients were divided into a dynamic clopidogrel resistant (DCR) group and a continuous Non clopidogrel resistance (NCR) group. Patients were randomly assigned to AC group (atorvastatin 40 mg/d + clopidogrel, 51 patients) and RC group (rosuvastatin 20 mg/d + clopidogrel, 47 patients). The patient’s platelet aggregation rate (PAR) was measured on visit 0 (baseline), visit 1 (1 week after clopidogrel alone treatment), and visits 2 to 4 (one, three, and 6 months after clopidogrel plus statins treatment). The platelet reactivity index (PRI) was assessed on visits 0, 2, and 4, and clopidogrel thiol metabolite (H4) levels was measured on visits 2 and 4. DNA sequencing was used to determine *CYP3A4*, *CYP2C9*, and *CYP2C19* genotypes in all patients.

**Results:** PAR, PRI, and H4 levels, DCR ratio, and the genotype frequencies of CYP2C9*3εC, CYP2C19*2εA, and CYP2C19*3εA of both groups were similar (*p* > 0.05). *CYP2C19εA* *2 and *3 were independent risk factors for DCR (*p* < 0.05).

**Conclusion:** Clopidogrel combined with atorvastatin does not affect platelet inhibition and does not increase the incidence of DCR. The incidence of DCR in the Chinese population is high and is related to *CYP2C19εA.*

## Highlights

1) The patient’s platelet aggregation rate, platelet reactivity index, and clopidogrel thiol metabolite levels of AC group (atorvastatin 40 mg/d + clopidogrel, 51 cases) and an RC group (rosuvastatin 20 mg/d + clopidogrel, 47 cases) were similar (*p* > 0.05).
2) Compared with the AC group, the dynamic clopidogrel resistant ratio of the RC group and the genotype frequencies of CYP2C9*3εC, CYP2C19*2εA, and CYP2C19*3εA were not significantly different (*p* > 0.05). *CYP2C19εA* *2 and *3 were independent risk factors for DCR (*p* < 0.05).
3) Clopidogrel combined with atorvastatin (CYP3A4-metabolized) does not affect platelet inhibition by clopidogrel and does not increase the incidence of DCR. The incidence of DCR in the Chinese population is high and is related to *CYP2C19εA.*

## 1 Introduction

Clopidogrel is a common antiplatelet agent and clinical trials have shown that long-term use of clopidogrel can effectively reduce serious cerebrovascular events (Levine et al., 2016; Amelia et al., 2019; Ma et al., 2019). However, due to individual differences, cardiovascular and cerebrovascular events still occur in some patients despite regular long-term use of clopidogrel, a phenomenon known as Clopidogrel resistance (CR) (Yi et al., 2017; Karaz´niewicz-Łada et al., 2012; Sofi et al., 2010; Yi et al., 2016; Zhou et al., 2013). The incidence of CR ranges from 16.8 to 21%, and can reach 44% in TIA and stroke. The results of our previous study showed that the incidence of CR in patients with ischemic stroke was 30.6% (Yi et al., 2017; Karaz´niewicz-Łada et al., 2012; Sofi et al., 2010; Yi et al., 2016; Zhou et al., 2013).

The incidence of clopidogrel resistance is high and there are many influencing factors (Yi et al., 2017; Karaz´niewicz-Łada et al., 2012; Sofi et al., 2010; Yi et al., 2016; Zhou et al., 2013). Related studies have shown that in addition to genetic factors, clopidogrel resistance is related to the basis of disease (atherosclerosis, diabetes) and drug combination (Yi et al., 2017; Karaz´niewicz-Łada et al., 2012; Sofi et al., 2010; Yi et al., 2016; Zhou et al., 2013). In the primary and secondary prevention of cardio-cerebrovascular stroke events, the combination of antiplatelet aggregation drugs and statins has become a fixed combination, and the combination rate is higher than that of antihypertensive drugs and hypoglycemic drugs (Yi et al., 2017; Karaz´niewicz-Łada et al., 2012; Sofi et al., 2010; Yi et al., 2016; Zhou et al., 2013).

Clopidogrel is a drug precursor that is oxidized by CYP450 line (mainly CYP3A4 and CYP3A5) into active metabolites, which irreversibly bind with ADP receptor P2Y12 located on platelets, thus playing an anti-platelet aggregation role (Levine et al., 2016; Ma et al., 2019). Statins, reductase inhibitors of hydroxymethyl glutaryl Coenzyme A (HMG-COA), It can effectively reduce low density lipoproteins (LDL) and Total cholesterol (TC), and reduce Triglyceride (TG) to a certain extent (Pelliccia et al., 2014a; Kei et al., 2016; Pan et al., 2017; Xingyang et al., 2018a; Xingyang et al., 2018b; Xi et al., 2019; Si et al., 2020). Clinical studies routinely divide statins into those metabolized by CYP3A4 (such as atorvastatin) and those metabolized by non-CYP3A4 (such as rosuvastatin).

Antiplatelet therapy that combines statins has become standard in patients with MACCE. In China, clopidogrel is often used, but physicians have not paid much attention to the drug–drug interaction between statins and clopidogrel due to the lack of Chinese data. Therefore, combination therapy with CI patients, using statins and clopidogrel, needs further exploration.

Static clopidogrel resistance refers to clopidogrel resistance that occurs after taking clopidogrel for 1 week; dynamic clopidogrel resistance refers to clopidogrel resistance that does not occur after taking clopidogrel for 1 week, and clopidogrel resistance occurs after continuing to take the drug. In a previous study, (Zhou et al., 2013) we reported that dynamic CR (DCR) may occur after CI, probably due to the atorvastatin–clopidogrel interaction. However, this was only an observation study, and the aim was not to compare the drug–drug interactions between different metabolized statins and clopidogrel. Therefore, it has been necessary to design a prospective study to identify the different drug–drug interactions between CYP3A4-metabolized and not CYP3A4-metabolized statins and clopidogrel in CI patients. This paper focuses on the dynamic changes of non-CYP3A4-metabolized and CYP3A4-metabolized statins and CR in patients with CI.

## 2.Subjects and Methods

### 2.1 Study Design

**FIGURE 1:**
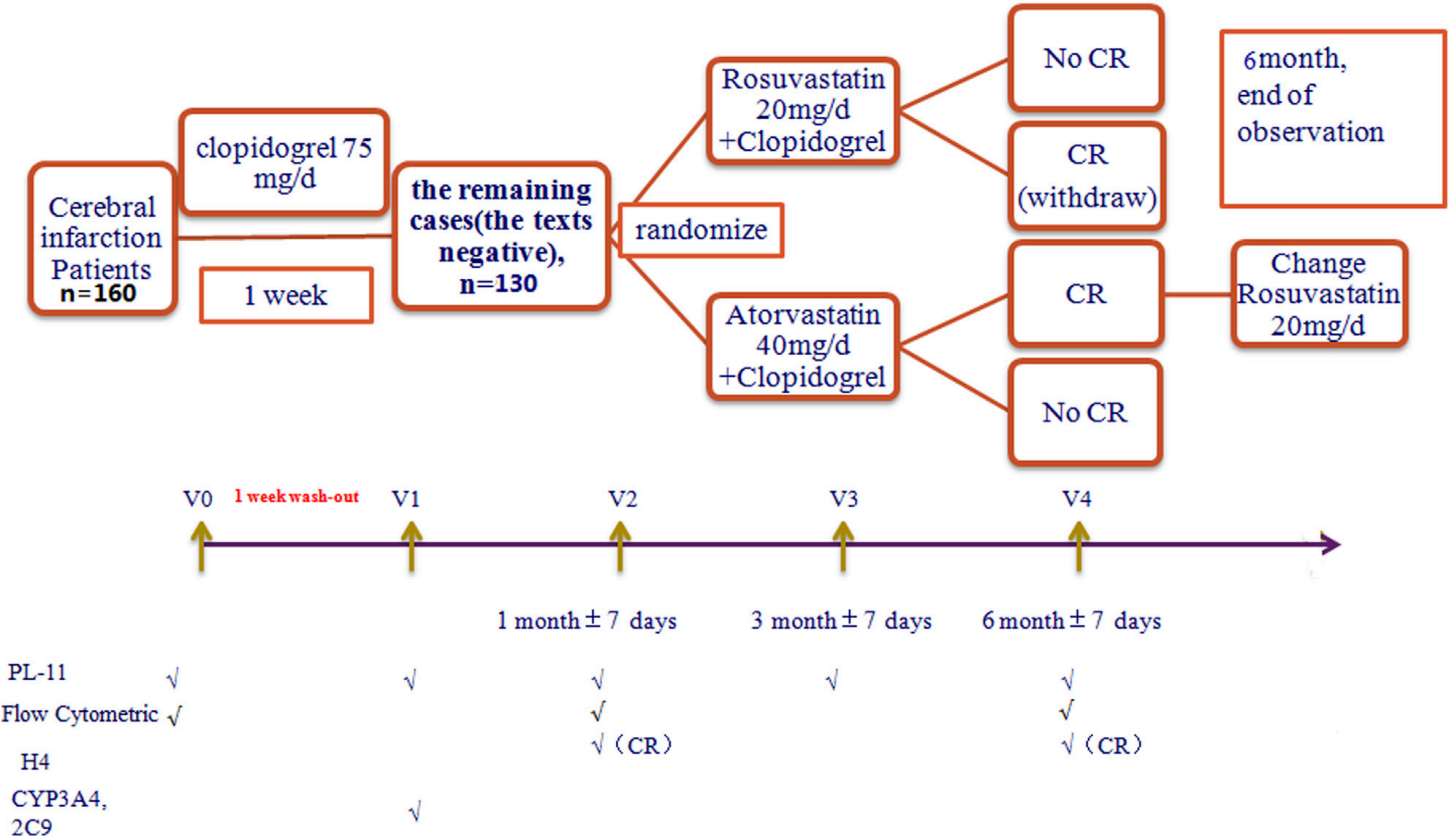
Study design.

This randomized, open-label, single-center study aimed to investigate the different drug–drug interactions between different metabolized statins and clopidogrel in patients with CI. Some research data (Yi et al., 2016; Pan et al., 2017; Xingyang et al., 2018a) has shown that static CR is mainly attributed to genetic polymorphisms, especially receptor gene CYP2C19. Our previous research also (Zhou et al., 2013) demonstrated that the DCR phenomenon occurs as well as static CR. Thus, in this study, static CR was excluded to minimize the influence of genetic factors on CR in CI. 160 Patients with CI received clopidogrel 75 mg/day (Sanofi). Their platelet aggregation function was tested using a PL series platelet function analyzer (PL-11) at baseline (pre-therapy) and 1 week after clopidogrel treatment, and the platelet aggregation rates (PARs) were calculated. The 30 patients who showed CR were eliminated. The 130 non-clopidogrel resistant (NCR) patients were randomly given either atorvastatin 40 mg/d (Pfizer) plus clopidogrel, or rosuvastatin 20 mg/d (AstraZeneca) plus clopidogrel in a 1:1 ratio, and were further divided into a DCR and a continuous NCR (CNCR) group, according to their PAR results at one, three, and 6 months. All the subjects were followed up for 6 months. If CR occurred with the co-administration of atorvastatin and clopidogrel (the AC group), the subject was switched to rosuvastatin. However, if CR occurred with the co-administration of rosuvastatin and clopidogrel (the RC group), the treatment was withdrawn, and the patients were given aspirin instead, and any major adverse cardiovascular and cerebrovascular events were recorded at 6 months (see Figure 1 for the study design). 130 Patients were followed up by telephone or a return visit to ensure compliance with the secondary prevention of CI guidelines in 2011. (Naylor, 2011).

#### 2.1.1 Subjects

All the subjects were recruited at the Third Affiliated Hospital of Guangzhou Medical University between March 2015 and September 2017. The genic and H4 data of all the subjects were completed in May 2018. During this period, 160 patients with various types of CI, who were stable after suffering an acute CI, were recruited. The clinical diagnosis of CI was made in accordance with standards established by the World Health Organization. (The World Health Organiza, 1988). One week after clopidogrel treatment, 30 cases (18.75%) showed CR and were eliminated from the study, and, thus, 130 cases with NCR remained. During the observation period, 2 cases (1.5%) withdrew due to an allergy, and 30 cases (23%) withdrew because of the financial burden or settlement abroad, so 98 cases (75%) completed all the observations.

A total of 130 patients with first or second onset of acute ischemic stroke hospitalized in the Department of Neurology of the Third Affiliated Hospital of Guangzhou Medical University from March 2015 to September 2017 were selected continuously, and 98 patients (75%) were finally observed. During the observation period, 2 patients (1.5%) withdrew from the observation due to drug allergy. Thirty patients (23%) withdrew from the study due to economic burden and settlement in other places. Inclusion criteria for the ischemic stroke group were as follows: the diagnosis was confirmed by computed tomography (CT) or 3.0T magnetic resonance imaging (MRI) in accordance with the world Health Organization standards. This study was approved by the Ethics Committee of the Third Affiliated Hospital of Guangzhou Medical University, and all patients or their families signed informed consent. 1) All patients with cerebral infarction met the criteria revised by the National Cerebrovascular Disease Conference. 2) Age >45 and <85; 3) NIHSS score ≤23; 4) Platelet count >150 × 109/L and <500 × 109/L; 5) Taking clopidogrel for the first time, or taking aspirin, dipyriddamole, clopidogrel and other antiplatelet aggregation drugs before but stopping for at least 2 weeks; 6) did not take statins or had stopped taking statins for 2 weeks; 7) The patient and his/her family have informed consent and can adhere to the medication and cooperate with the visitors. Exclusion criteria 1) Patients allergic to clopidogrel, rosuvastatin and atorvastatin; 2) Patients with NIHSS score >23 or NIHSS scale problem 1A score of 2 or above are excluded; 3) Patients with atrial fibrillation and ventricular septal defect; 4) Severe damage of heart, liver and kidney function or complicated with hemorrhage, tumor, immune system, respiratory system and other diseases; 5) Patients who have recently taken a proton pump inhibitor. 6) Recent major surgery or serious external injuries; 7) Patients who cannot receive regular outpatient follow-up or patients with poor compliance.

The protocol was approved by the Ethics Committee of the Third Hospital affiliated to Guangzhou Medical University. All the participants provided written informed consent prior to participating in the study.

#### 2.1.2 Platelet Function

Blood samples were collected using the double-syringe technique, in which the first 4 ml of blood was discarded to avoid spontaneous platelet activation. The platelet function was measured using a PL-11 analyzer (SINNOWA Medical Science &Technology Co., Nanjing, China) (Xiao et al., 2019) and a flow cytometer (BD Biosciences, USA) (Karina et al., 2019). The PL-11 analyzer system calculated the maximal platelet aggregation rate (PAR) according to the following formula: PAR max (%) =(the baseline platelet count—the lowest platelet count)/the baseline platelet count × 100%. CR was defined as being present when the maximal PAR was ≥55%. A platelet reactivity index (PRI) was calculated using MFIc in the presence of PGE1 alone (PGE1) or PGE1 + ADP according to the following calculation: PRI = [(MFIc PGE1—MFIc (PGE1 + ADP) ]/MFIc PGE1 x 100. CR was defined as: post therapy PRI > (pretherapy mean PRI—at 2 standard deviation).

Principle and method of pl-11 multiparameter platelet multifunction analyzer to detect platelet aggregation function.

##### The Principle of Measurement

Pl-11 multiparameter Platelet multifunction analyzer mainly measures Platelet aggregation function according to SPCM (continuous Counting Method). The instrument continuously counted the number of platelets in patients before and after the addition of ADP reagent, and evaluated the platelet aggregation function by comparing the change of platelet number and the rate of platelet decline before and after the addition of ADP reagent. MAR Max Aggregation Ratio (%) = (original platelet count -- number of aggregated platelets)/original platelet count ×100%.

##### Operation Steps

1) Open the power button of pl-11 multiparameter platelet multifunction analyzer, and the instrument will automatically enter the state of cleaning and self-test for about one minute; After the test is complete, wait until the word “Ready” is displayed at the top of the screen to start the test.
2) Turn on the power button of the thermostatic mixer and adjust the temperature to 37°C.
3) Sample preparation: during the test, the indoor temperature should not be lower than 22°C, and the test can be started only after mixing samples with a constant temperature blender for 5–10 min, and the thermostat should be set to 25°C. New blood was extracted with 1:9 anticoagulant tube of citrate, and the test was completed within 2 h after blood collection. When transferring the sample, you need to handle it gently and do not shake it forcibly.
4) Start testing: The samples shaken by the constant temperature blender are divided into 0.5 ml sample tubes with pipette gun, and then open the sample door of the instrument and place the sample tube in the sample position. Press the instrument start button and the instrument starts to operate. The multiparameter platelet analyzer will automatically perform two platelet counts, after which the screen will appear indicating the addition of ADP attractant. At this time, the sample site door will be opened again, and 40 ul ADP attractant will be added into the sample tube with a micropipette gun. Attention should be paid to this operation: the sample tube should not be taken out to add inducer. After adding the post-inducer on the tip of the micropipette gun, it should be sucked back and forth 2–3 times to make the inducer fully mixed with the blood sample, and the tip of the micropipette gun should be kept below the blood sample when it is sucked back and forth. The preceding procedure needs to be completed within 1 min. After adding inducer, close the side door, press [OK] button, and then press no. 1 button, the instrument will automatically measure 3 times, then the instrument will automatically stop, and the test results will be displayed on the screen. When the words “Waiting for test” and “Ready” are displayed again at the top of the screen, the next test can be performed.

#### 2.1.3 The Assessment of Clinical Characteristics

After admission, the patient’s gender, age, and blood pressure were recorded, and the patient’s past history, personal history, medication history and family history were carefully questioned. All patients received the National Institutes of Health Stroke Scale (NIHSS) score immediately after admission, and underwent brain CT or MRI examination on the same day. Blood routine examination, coagulation routine examination, biochemical routine examination, blood lipid routine examination, hBA1C and blood glucose examination were performed the next day. Platelet aggregation rates were evaluated before clopidogrel administration (interview 0), 1 week after clopidogrel administration (interview 1), 1 month after statin administration (interview 2), 3 months after statin administration (interview 3), and 6 months after statin administration (interview 4).

All patients were monitored with cranial magnetic resonance imaging scans and routine blood tests (platelet counts, international standardized ratios, glucose and lipid analyses). Hypertension was defined as systolic blood pressure ≥140 mmHg and/or diastolic blood pressure ≥90 mmHg, and/or the use of antihypertensive drugs. Diabetes was defined as fasting blood glucose ≥7.0 mmol/L, or hemoglobin a1c ≥ 6.0% and/or a history of diabetes. Hyperlipidemia was defined as total cholesterol ≥5.0 mmol/L and low density lipoprotein ≥3.5 mmol/L.

#### 2.1.4 Genetic Analysis

**FIGURE 2:**
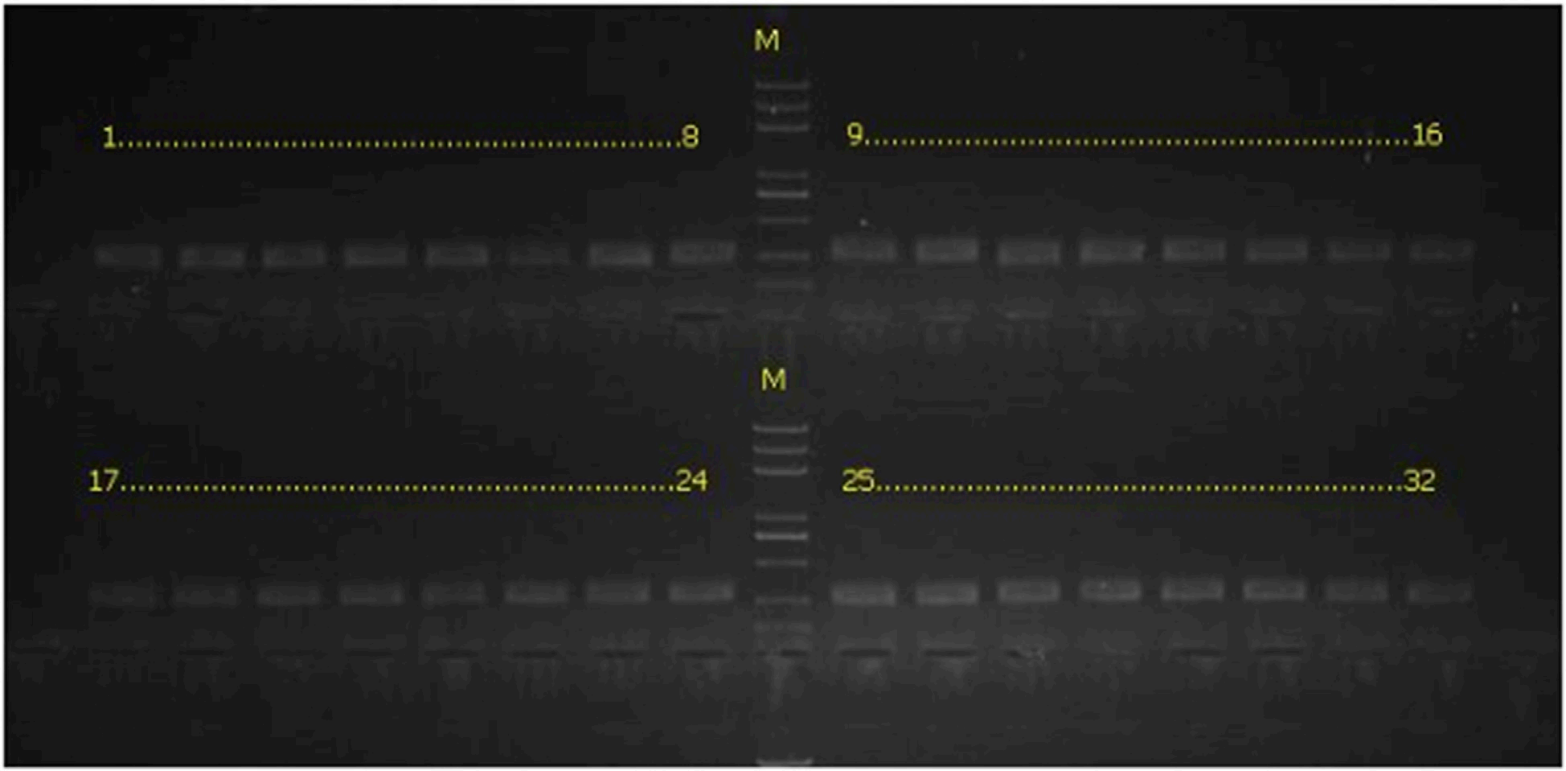
PCR amplification products map.

All subjects were genotyped for CYP3A4 receptor genes, CYP2C9, and CYP2C19. The reference sequence was found in the National Center for Biotechnology Information, and DNA was extracted from the whole blood samples with a TIANamp Blood DNA Kit (Sigma Corporation, USA) according to the manufacturer’s instructions. The primer sequences can be seen in Table 1 and the PCR amplification products map in Figure 2.

**TABLE 1:** The primers of genes.

Genes	Primers	Product length
CYP2C9*2	F: CCCTCCTAGTTTCGTTT	
rs1799853	R:GCACAATCATGCCTGTA	376 bp
CYP2C9*3	F:TGTCTTATCAGCTAAAGTCCAGG	286 bp
rs1057910	R:CCCGGTGATGGTAGAGGTTT	
CYP2C19*2	F:AAAGCAGGTATAAGTC	494 bp
rs4244285	R:CATCCGTAGTAAACAC	
CYP2C19*3	F:CACTTTCATCCTGGGCTGTG	292 bp
rs4986893	R:TCTTTTCCAGATATTCACCCCAT	
CYP3A4*3	F:CTACTAGTTGAGGGGTGGCC	383 bp
rs4986910	R:TCTTTGGCCCAGAGAACAAA	

#### 2.1.5 The Determination of Clopidogrel Thiol Metabolite

Five mL of blood was drawn from patient 1 and 6 months after clopidogrel administration into vacuum systems containing EDTA-K (Yangpu Medical Technology Company, China). Clopidogrel thiol metabolite H4 (H4) was measured by (LC-MS/MS) (Karaźniewicz-Łada et al., 2012) (Figure 6).

Chromatography-mass spectrometry procedures were as follows.

1)Setting of blood sample collection time: Blood samples were collected 1 month and 6 months after clopidogrel administration (75 mg or 300 mg).
2)Preparation and use of stabilizer. Preparation: alkylation agent MBP(500 mm.L-1): ethyleneglycol solution (20 ml)+MBP(2.2907 g), fully dissolved, refrigerated at 4 °C for reserve. Use: Blood + stabilizer = 200:1 (V: V), [final concentration of MBP = 2.5 mm.L-1], that is, add 25 ul of 500 mm.L-1 stabilizer MBP per 5 ml blood.
3)Specimen pretreatment (sample collection and preservation). Preliminary treatment: The blood samples with stabilizers were immediately centrifuged, 1620 *g* × 10 min X 4°, and the supernatant and cell precipitate were collected respectively. The supernatant was used for GC/MS analysis, and the cell precipitate was used for gene polymorphism analysis (extracting RNA or DNA for PCR). The supernatant isolated from each sample was separated into EP tubes with 0.5 ml for each tube. Divide into several tubes, mark and store at minus 80° for later use.
4)Sample processing before machine (mass spectrometry analysis before processing). Take 25 μl of sample standard solution or supernatant of sample to be tested using EP tube, add 25 μl of internal standard solution, and mix well. Add 250 μl of blank plasma and mix well. Next, 450 μl of cold acetonitrile is added to precipitate the protein. Swirl for 5 min, then centrifuge 22570 *g* × 20° for 10 min, take the supernatant, filter with mini filter, collect 100 ul of filtrate, dry under vacuum 40°, obtain dry powder, use 200 μl of mobile phase liquid (A + B 50: 50, V: V) dissolved, the suspension was centrifuged again at 22570 g × 200° for 10 min, and 25 μl of the supernatant was absorbed for sample injection and GC-MS analysis.
5)Chromatographic and mass spectrometry analysis. Chromatographic conditions: Chromatographic column was Zorbax Plus C18 Column (100 mm/2.1 mm, 3.5 μm, Agilent Technologies, USA). Mobile phase was acetonitrile (containing 0.1% formic acid): water (containing 0.1% formic acid) (9:1, V/V). Washing balance was as follows: The gradient was as follows:0–7 min linear from 42 to 90% B, Return from 90 to 42%B and the post time of 5 min with 42%B for column Slides bration. The flow rate was 350 μl min^−1^. Sample volume was 25 μl. The column temperature was 40°C. The analysis time was 3.2 min.
6)Mass spectrometry conditions: Ion source:ESI + electrospray ionization; Detection method: positive ion mode; Scanning methods: multiple response monitoring (MRM); Spray gas: N2 (40 psi); Solvation gas: N_2_ (10 L/min); Solvent removal gas flow rate: 650 L h^−1^; Solvent temperature: 300°C; Ion source temperature: 120°C; Collision chamber gas: N_2_
7) preparation of mother liquor
  1)Preparation of the original clopidogrel hydrogen sulfate reserve solution: weigh 1 mg clopidogrel hydrogen sulfate, add 1 ml acetonitrile to make its final concentration 1.0 mg/ml, and store in the refrigerator at −20°C for later use.
  2)Preparation of active metabolic derivative of clopidogrel (MP-H4) reserve liquid: accurate respectively. Weigh 1 mg MP-H4, add 1 ml acetonitrile to make its final concentration 1.0 mg/ml, and freeze it in the refrigerator at −20°C for later use.
  3)Preparation of internal standard piroxicam storage and distribution solution: weigh 1 mg piroxicam, add 1 ml acetonitrile to dissolve it, make its final concentration 1.0 mg/ml, and freeze it in the refrigerator at −20° for reserve.
8) Working fluid preparation
  1)Preparation of clopidogrel hydrogen sulfate working solution (0.1 mg/ml = 100 ug/ml = 100000 ng/ml): 1.0 mg/ml mother solution diluted 10 times with L acetonitrile.
  2)Preparation of clopidogrel active metabolic derivative (MP-H4) working solution (0.1 mg/ml = 100 ug/ml = 100000ng/ml): 1.0 mg/ml mother solution diluted 10 times with L acetonitrile.

#### 2.1.6 Statistical Analysis

Statistical analysis was performed using the SPSS version 22.0 for Windows (SPSS Inc., USA). Continuous variables, presented as means and standard deviations (mean ± SD), were tested using Student’s t test. Categorical variables were compared using the Pearson chi-square test. PAR was tested using the general linear model-repeated measurement. H4 was tested using the Sphericity Assumed. Analysis of variance (ANOVA) was used to compare platelet function and H4 changes. Correlation between the genotype and allele frequency distribution differences and the CR were analyzed using multivariate regression models. Multivariable logistic regression analysis analyzed the relationship between DCR and risk factors. *p* < 0.05 was statistically significant.

## 3 Results

### 3.1 The Comparison of Patient Demographics

Among the original 160 subjects with CI, 30 cases (18.75%) where CR occurred were eliminated 1 week after the clopidogrel treatment had begun, and so 130 cases with NCR remained, with 98 cases completing the six-month follow-up. Thirty-two patients (24.5%) were excluded because of inadequate laboratory monitoring; 14 from the AC group and 18 from the RC group. Dynamic observation was continued in 51 AC cases and in 47 RC cases. There was no significant difference between the AC and RC groups with respect to general demographic information (*p* > 0.05). Of the 51 AC cases, 32 (62.75%) were male and the mean age was 67.69 ± 8.88 years, and of the 47 cases in the RC group, 32 (68.09%) were male and the mean age was 65.66 ± 11.80 years. The AC group and RC groups were comparable with respect to the incidence of co-existing hypertension (68.62 vs 76.60%, respectively), DM (37.25 vs 27.70%) and hyperlipidemia (37.25 vs 36.17%) (all *p* > 0.05, see Table 2).

**TABLE 2:** Clinical characteristics of comparison between AC and RC in CI patients.

Characteristic	ACgroup (*n* = 51)	RCgroup (*n* = 47)	Chi-square or t value	*p* Value
Age, years	67.69 ± 8.88	65.66 ± 11.80	0.967	0.336
Male [n (%)]	32 (62.75)	32 (68.09)	0.308	0.579
Smoke [n (%)]	16 (31.37)	12 (25.53)	0.409	0.523
NIHSS score	4.65 ± 3.18	5.05 ± 2.36	0.813	0.510
Hypertension, n (%)	35 (68.62)	36 (76.60)	0.778	0.378
Diabetes mellitus, n (%)	19 (37.25)	13 (27.70)	1.024	0.312
Hyperlipidemia, n (%)	19 (37.25)	17 (36.17)	0.012	0.911
Calcium antagonists, n (%)	24 (47.06)	22 (46.81)	0.001	0.980
Beta blockers, n (%)	17 (33.33)	15 (31.91)	0.022	0.881
ACEI/ARB, n (%)	15 (29.41)	17 (36.17)	0.508	0.476
Diuretics, n (%)	9 (17.65)	12 (25.53)	0.903	0.342
Sulfonylureas, n (%)	9 (17.65)	10 (21.28)	0.206	0.650
Glycosidase, n (%)	6 (11.76)	6 (12.77)	0.023	0.880
Biguanides, n (%)	6 (11.76)	7 (14.89)	0.208	0.648

**AC =** atorvastatin; **RC =** rosuvastatin; ACEI/ARB = angiotensin inhibitors.

### 3.2 The Comparison of Platelet Function

Statistical PAR with PL-11 was analyzed using Mauchly’s Test of Sphericity at each visit of the AC and RC groups. It was concluded that since *p* < 0.05, there was a lack of sphericity, so in this study statistical PAR was tested using the Greenhouse–Geisser coefficient correction**.** Comparisons within groups and between groups were performed using the General Linear Model of ANOVA. The AC group and RC group were comparable with respect to PAR at each visit, and there was no significant difference (f = 0.049, *p* = 0.825), suggesting that non-CYP3A4-metabolized and CYP3A4-metabolized statins were not related to DCR. There was a significant difference within groups with respect to PAR at each visit (f = 10.823, *p* = 0.000). At Visit 0, PAR was 40.57 ± 8.95% for the AC group and 40.79 ± 8.35% for the RC group, compared with 33.91 ± 14.11% vs 33.73 ± 11.53% at Visit 1, respectively (all *p* < 0.05, see Table 3). However, prior to taking clopidogrel, and at 1 week, 1 month, 3 months, and 6 months after treatment began, the PAR was similar in the AC and RC groups (all *p* > 0.05, see Table 3).

**TABLE 3:** Comparison of PAR in different time periods for AC and RC in CI patients.

PAR(χ±S,%)	ACgroup (*n* = 51)	RCgroup (*n* = 47)
Visit 0	40.57 ± 8.96	40.79 ± 8.35
Visit 1	33.91 ± 14.11*	34.20 ± 11.47*
Visit 2	31.01 ± 14.48*	30.55 ± 12.79*
Visit 3	31.48 ± 13.67*	30.90 ± 12.12*
Visit 4	33.75 ± 16.04*	35.98 ± 16.34*

PAR = platelet aggregation rates; Visit 0 = Pretherapy; Visit 1 = 1 week posttreatment clopidogrel; Visit 2 = 1 month posttreatment clopidogrel plus statin; Visit 3 = 3 months sposttreatment clopidogrel plus statin; Visit 4 = 6 months posttreatment clopidogrel plus statin; AC = atorvastatin; RC = rosuvastatin; *p < 0.01 compared with Visit0.

Platelet function was analyzed using the flow cytometric assessment of VASP phosphorylation at Visit 0, 2, and 4 in the AC and RC groups. The analysis of statistical PRI was done in the same way as for statistical PAR. It was found that the PRI of the two groups was similar at each visit (f = 0.186, *p* = 0.667), but there was a significant difference within groups with respect to PRI at each visit (f = 294.087, *p* = 0.000). At Visit 0, it was 71.02 ± 10.40% for the AC group vs 71.40 ± 10.74% for the RC group (*p* > 0.05). In the follow-ups at 1 month (Visit 2) and 6 months (Visit 4), the PRIs of the two groups had decreased significantly from Visit 0. Those of the AC group were 29.23 ± 7.91% (Visit 2) vs. 39.29 ± 18.44% (Visit 4), those of the RC group were 28.21 ± 7.75% (Visit 2) vs 37.85 ± 1 8.67% (Visit 4), (*p* < 0.05, see Table 4).

**TABLE 4:** Comparison of PRI in different time periods for AC and RC in CI patients.

PRI (‾χ±S, %)	ACgroup (*n* = 51)	RCgroup (*n* = 47)
Visit 0	71.02 ± 10.40	71.40 ± 10.74
Visit 2	29.23 ± 7.91*	28.21 ± 7.75*
Visit 4	39.29 ± 18.44*	37.85 ± 18.67*

PRI = platelet reactivity index; Visit 0 = Pretherapy; Visit 2 = 1 month posttreatment clopidogrel plus statin; Visit 4 = 6 months posttreatment clopidogrel plus statin; AC = atorvastatin; RC = rosuvastatin; *p < 0.01 compared with Visit0.

### 3.3 The Comparison of H4

The H4 was unaltered with either atorvastatin or rosuvastatin was added to the treatment with clopidogrel (AC: 36.85 ± 11.94 ng/ml vs. RC: 34.73 ± 12.06 ng/ml at Visit 2 [*p* = 0.384] and AC: 22.76 ± 10.24 ng/ml vs RC: 20.60 ± 7.56 ng/ml at Visit 4 [*p* = 0.241]). However, the H4 was significantly different in the DCR and CNCR groups (DCR: 27.63 ± 10.74 ng/ml vs. CNCR: 38.21 ± 11.31 ng/ml at Visit 2 [*p* = 0.0001] and DCR: 17.98 ± 8.26 ng/ml vs. CNCR: 22.81 ± 9.06 ng/ml at Visit 4 [*p* = 0.027]). As expected, H4 was decreased in all patients at Visit 2 compared with Visit 4 (*p* < 0.05, see Table 5).

**TABLE 5:** Comparison of H4 in different time periods in CI patients.

Group	Visit 2 H4	Visit 4 H4
	(‾χ±S, ng/ml)	(‾χ±S, ng/ml)
DCR (*n* = 22)	27.63 ± 10.74	17.98 ± 8.26*
CNCR(*n* = 76)	38.21 ± 11.31	22.81 ± 9.06*
T value	3.906	2.244
*p* value	0.0001	0.027
AC (*n* = 51)	36.85 ± 11.94	22.76 ± 10.24*
RC (*n* = 47)	34.73 ± 12.06	20.60 ± 7.56*
T value	0.874	1.180
*p* value	0.384	0.241

H4 = clopidogrel thiol metabolite; Visit 2 = 1 month posttreatment clopidogrel plus statin; Visit 4 = 6 months posttreatment clopidogrel plus statin; DCR = dynamic clopidogrel resistance; CNCR = continuous none clopidogrel resistance; AC = atorvastatin; RC = rosuvastatin; *p < 0.01 compared with Visit2.

### 3.4 The Analysis of Genotypes for CYP3A4, CYP2C9, and CYP2C19

No cases of DCR were observed at 1 month and 3 months, but 22 cases had occurred at 6 months. The incidence of DCR was similar in the AC group and RC subgroups (12 vs 10, respectively, *p* = 0.789). The 12 AC patients were switched to rosuvastatin in combination with clopidogrel for the treatment of DCR, but the PAR (60.67 ± 5.13 vs. 59.35 ± 3.64, *p* = 0.224) had not decreased, 1 week after changing treatment, and they were still CR.

**FIGURE 3:**
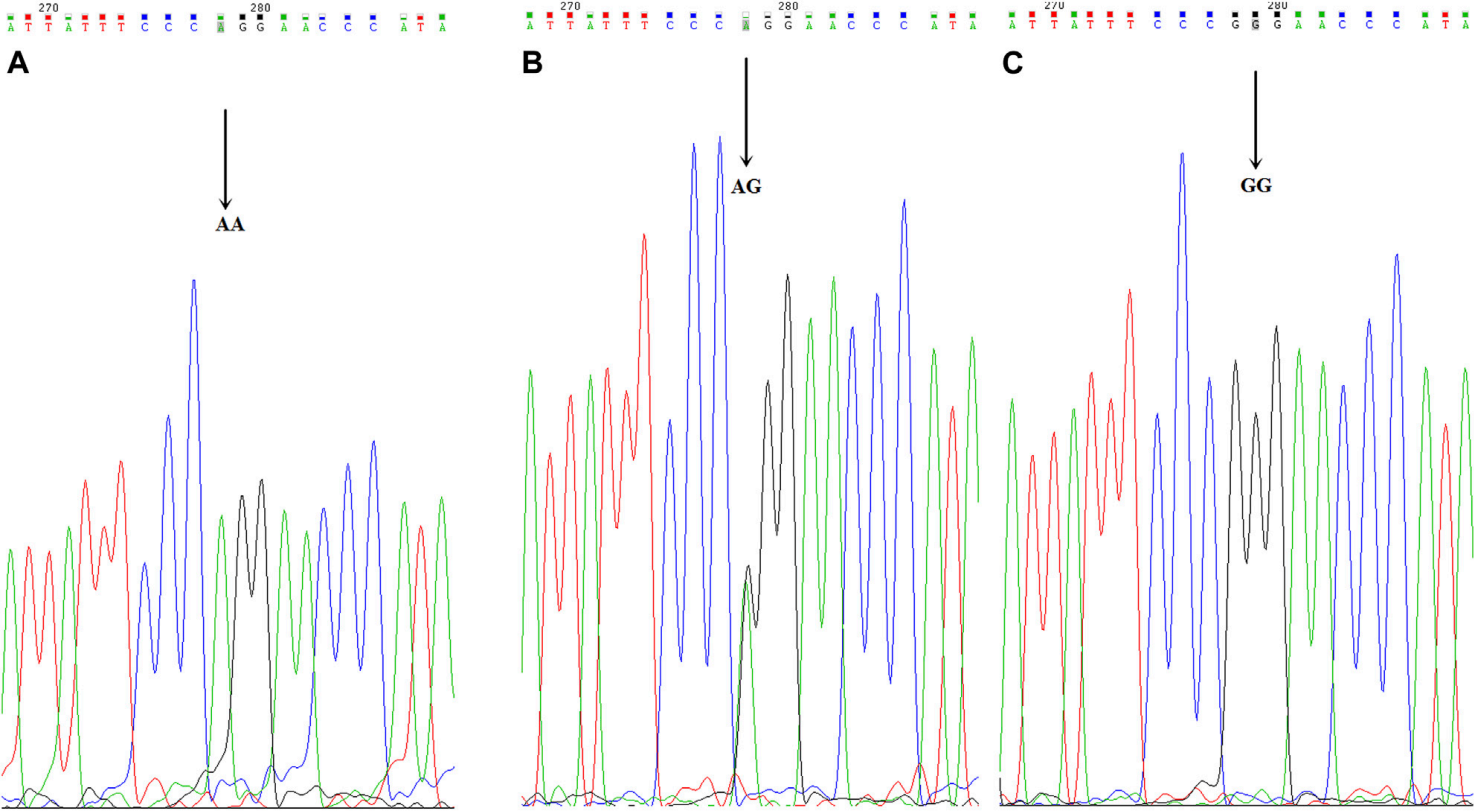
CYP2C19*2 mutant allelic variant map. ABC is the mutation sequence, and the arrow shows the mutation site (AA/AG/GG), where AA/AG is the mutant gene.

**FIGURE 4:**
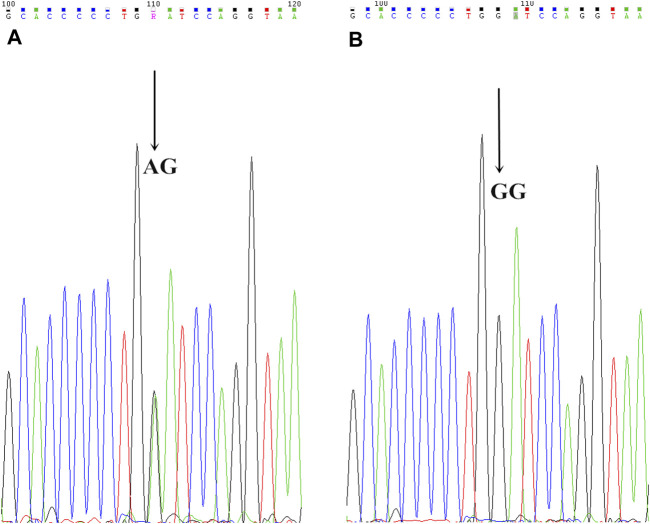
CYP2C19*3 mutant allelic variant map. AB is the mutation sequence, the arrow is the mutation site (AA/GG), where AG is the mutant gene.

**FIGURE 5:**
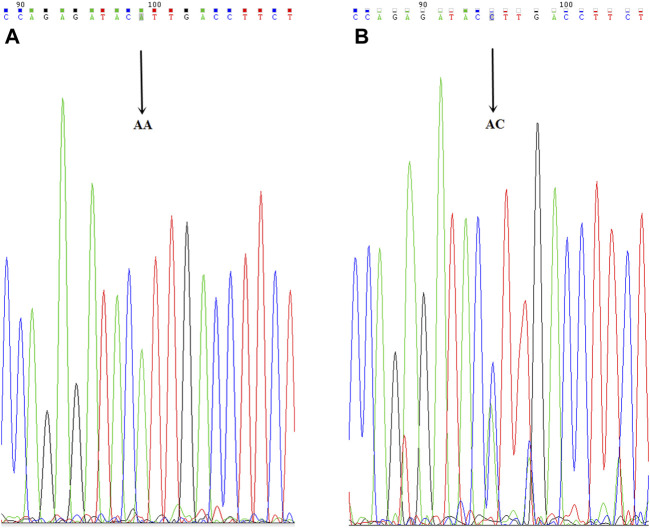
CYP2C9*3 mutant allelic variant map. AB is the mutation sequence, and the arrow is the mutation site (AA/AC), where AC is the mutant gene.

The analyses of the CYP3A4, CYP2C9, and CYP2C19 genotypes are shown in Table 6. The CYP2C9*3 genotype was found in four AC patients, and two RC cases (*p* = 0.548). The CYP2C9*3 mutant allelic variant can be seen in Figure 5. The CYP2C9*3 wild-type (*a/*a) occurred with a frequency of 93.88% (92/147), while 6.12% (6/98) were heterozygous for CYP2C9*3 (*a/*c), and none of the CYP2C9*3 was homozygous (*c/*c). The CYP2C9*3 εC in DCR was higher than CNCR (22.73 vs 1.32%, respectively, *p* = 0.0002). The AC and RC groups were comparable with respect to CYP2C19*2 and *3, there being no significant differences (CYP2C19*2 for 45.09 vs 46.81% respectively, [*p* = 0.865] and CYP2C19*3 for 9.80 vs 4.26% respectively [*p* = 0.287]). In 98 patients, 53 patients (54.08%) were wildtype homozygous for the CYP2C19*2 (*G/*G) gene, and 91 patients (92.86%) for the CYP2C19*3 gene, while 44 patients (44.90%) were heterozygous for CYP2C19*2 (*A/*G), and 7 patients (7.14%) for CYP2C19*3, and 1 patient (1.02%) was homozygous (*A/*A) for CYP2C19*2, and no one for CYP2C19*3. The CYP2C19 εA was significantly higher in DCR than CNCR (CYP2C19*2 for 81.82 vs 35.53% [*p* = 0.001], and CYP2C19*3 for 18.18 vs 3.95% [*p* = 0.0224]) (see Table 6). Finally, none of the CYP3A4 (*T/*T) and CYP2C9*2 (*C/*C) was a mutant allelic variant. (For CYP2C19*2 mutant allelic variant see Figure 3, and CYP2C19*3 mutant allelic variant see Figure 4).

**TABLE 6:** Analysis of genotypes for CYP3A4, CYP2C9 and CYP2C19 in CI patients.

Group	CYP2C9*3 [n (%)]	CYP2C19*2 [n (%)]	CYP2C19*3 [n (%)]
DCR (*n* = 22)	5 (22.73)	18 (81.82)	4 (18.18)
CNCR(*n* = 76)	1 (1.32)	27 (35.53)	3 (3.95)
T value	13.609	14.723	5.212
*p* value	0.0002	0.0001	0.0224
AC (*n* = 51)	4 (7.84)	23 (45.09)	5 (9.80)
RC (*n* = 47)	2 (4.26)	22 (46.81)	2 (4.26)
T value	0.548	0.029	1.135
*p* value	0.459	0.865	0.287

DCR = dynamic clopidogrel resistance; CNCR = continuous none clopidogrel resistance; AC = atorvastatin; RC = rosuvastatin

**FIGURE 6:**
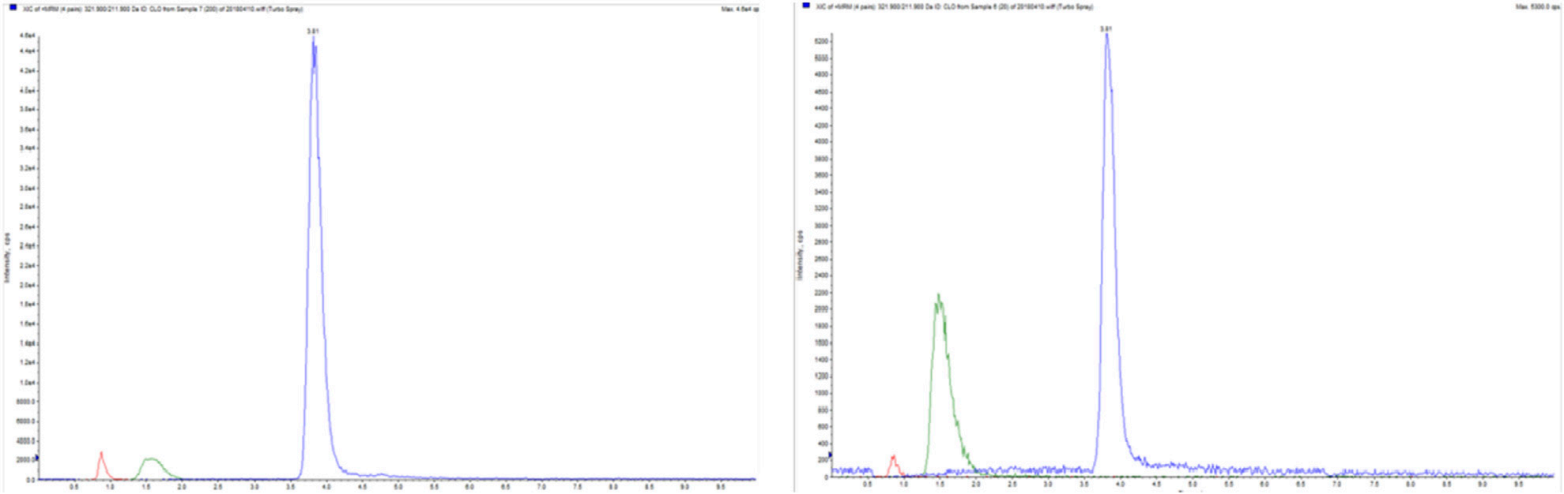
Clopidogrel thiol metabolite (H4) map.

### 3.5 Adverse Events

The primary safety endpoint occurred in 10 (10.20%) of the study population within the 6 months. There were 3 lacunar cerebral infarctions (2 in the AC group, 1 in the RC group), 3 elevated liver enzyme cases (2 AC, 1 RC), and 4 elevated muscle enzyme cases (2 AC, 2 RC). There was no significant difference between the AC and RC groups with respect to these adverse events (*p* > 0.05).

## 4 Discussion

The results of this study concerning statins are in line with previous research findings. (Gulec et al., 2005; An et al., 2019; Takashi et al., 2020). Lau et al. reported that atorvastatin could reduce clopidogrel activation by 90% and reduce the clopidogrel antiplatelet effect, attracting wide attention. As with our findings, observational studies by Ke A et al. and Gulec et al., Gulec et al., 2005; An et al., 2019) found that clopidogrel combination with atorvastatin increased adverse cardiac events in coronary stent implantation patients compared with vastvastatin. Other studies (Blagojevic et al., 2009; Wenaweser et al., 2010; Tirkkonen et al., 2013; Pelliccia et al., 2014b; Jin et al., 2017) did not confirm the effect of cyp3a4 metabolism on the clopidogrel antiplatelet effect. Even considering our findings, fewer studies have been reported on the dynamics of clopidogrel resistance, and practical differences need to be further explored. This study aimed to evaluate the difference in dynamic clopidogrel resistance between rosuvastatin 20 mg/d and atorvastatin 40 mg/d in patients with cerebral infarction. This study provided patients with a free platelet aggregation test using PL-11 and flow cytometry.

In this study, clopidogrel was used in combination with rosuvastatin or atorvastatin, and the PAR showed a gradually declining trend from visit 0 to visit 4. It was also found that the AC group and RC group were comparable with respect to the PAR at each visit, and there was no significant difference. It was concluded that clopidogrel and atorvastatin or rosuvastatin are safe when used in combination for the treatment of CI patients.

VASP (Karina et al., 2019) is an intracellular platelet protein that is non-phosphorylated at the basal state. It was also observed that for clopidogrel in combination with atorvastatin or rosuvastatin there was no reduction in the platelet inhibition of clopidogrel and no increase in platelet activity. The PRI was similar in the RC group and RC subgroups at visits 0, 2 and 4. This result also suggested that CYP3A4-metabolized statins are not related to DCR.

Clopidogrel (Dansette et al., 2012; Laizure et al., 2013; Zhu et al., 2013) is a predrug that requires biotransformation of the liver to play a pharmacological role. The active metabolites of clopidogrel contain a sulfate group that irreversibly binds to free cysteine in the P2Y12 receptor and prevents the activation of ADP. In humans, most drugs (85–90%) are metabolized as carboxylic acid metabolites through carboxylate estase, the main metabolite of circulation in the blood. Although this metabolite is inactive, for years, plasma determination in plasma has been used to indirectly study the pharmacokinetics of clopidogrel with only a small fraction of the drug mediated two-step activation process by cytochrome P450 (CYP450). This hydrolysis pathway competes with the formation of active metabolites catalyzed by the liver CYP450 enzymes. Clopidogrel is metabolized by CYP450 enzyme to form inactive intermediates 2-oxo-clopidogrel, and in the human body is further metabolized by CYP450 enzyme to form three sulfate metabolites, of which only H4 metabolites have antiplatelet effects.

Some statins are similar to clopidogrel, metabolized by CYP3A4, while others do not by CYP3A4 (Gulec et al., 2005; An et al., 2019; Takashi et al., 2020), so clopidogrel-statatin interactions can be analyzed by H4 concentrations in the blood. This study used proven HPLC-mass spectrometry combination techniques to determine H4. This study found that there was no change in the serum concentrations of clopidogrel active metabolites when atorvastatin or reassuvastatin was added in clopidogrel therapy. This study showed that neither atorvastatin nor resuvastatin altered clopidogrel-mediated platelet aggregation inhibition.

A large number of studies (Hou et al., 2014; Serbin et al., 2016; Yang et al., 2016; Danielak et al., 2017; Dorota et al., 2017) suggest that a variety of genetic and non-genetic factors may affect clopidogrel resistance phenomena. CYP3A4 and CYP2C19 are the most important CYP450 isozymes that activate clopidogrel. Most notably, CYP2C19 deficiency alleles such as * 2 and * 3 are associated with poor clopidogrel reactions, lower concentrations of H4 metabolites and higher clinical adverse events in nongenetic factors, while statiins are associated with adverse cardiovascular events Furthermore, lower active metabolites exposure was observed when cyp3a4 metabolized statin (especially atorvastatin and risuvastatin) are administered with cyp3a4 metabolized statin (atorvastatin and risuvastatin) Clopidogrel. Other genetic polymorphisms that occur in gene sequences are also considered to be a potential factor for a variable clopidogrel reaction. As shown in the literature, CYP2C9 genotypes may be related to the lower expression of the parent drug (Danielak et al., 2017) for the DNA sequence analysis of CYP3A4, CYP2C9, and CYP2C19. CYP2C9 was found that C and CYP2C19 εA in DCR were higher than CNCR, suggesting that the presence of CYP2C9 and CYP2C19 alleles reduces platelet inhibition of clopidogrel and increases the incidence of DCR and clinical adverse events. Similarly, in a previous study, (Zhou et al., 2013) showed that CYP2Y19εA was a risk factor for CR. However, the AC and RC groups are comparable in CYP2C9 and CYP2C19, with no significant differences. Non-cyp3a4 is considered to be a mutant allele variant.

According to our data, clopidogrel combined with atorvastatin or vastvastatin does not affect platelet inhibition of clopidogrel and does not increase the incidence of DCR, so cyp3a4 nonmetabolic and cyp3a4 metabolic statins are not associated with DCR. One explanation for this effect may be that drug interactions are affected by many factors. Clopidogrel is metabolized by CYP450, with multiple isoenzymes involved, such as CYP1A2, CYP2C9, CYP2D6, and CYP3A5, but the two most important isoenzymes, CYP3A4 and CYP2C19, are not involved. (Serbin et al., 2016; Yang et al., 2016). The combination of CYP3A4 and CYP2C19 inhibitors may not be sufficient to affect the metabolism of clopidogrel. Furthermore, statin doses used clinically may not reach the saturation concentrations of CYP3A4 and CYP2C19 and therefore do not produce competitive inhibition, which may be part of the results of our study.

This study also showed that when DCR occurred after CI, the incidence of DCR resistance was 22.45%, which was related to CYP2C9 εC and CYP2C19 εA. At week 1, there were 30 cases (18.75%) of static CR, compared with our previous study, (Zhou et al., 2013) where the incidence of staticCR was 28.6%, the total CR (static CR and DCR) rate being 38.8%. CHANCE’s (Wang et al., 2013) reported that the benefits of dual antiplatelet therapy come mainly from patients in CR with poor metabolizer of CYP2C19 gene. In addition to the use of aspirin, some patients treated with clopidogrel did not show an adequate antiplatelet response in dual antiplatelet therapy. Some studies (Fohner et al., 2013; Sang et al., 2017; Zhong-ling et al., 2018) have shown that CYP2C19 and CYP2C9 genes are the dominant metabolic enzymes in the CYP450 metabolic enzymes of eastern populations, while the CYP2D6 gene is the dominant metabolic enzyme in western populations. It may be that the differences in genetic metabolic enzymes can lead to significantly higher static CR and DCR in eastern populations. The Prevention Regimen for Effectively Avoiding Second Strokes and Clopidogrel Versus Aspirin in Patients at Risk of Ischemic Events (Steering Committee, 1996; Sacco et al., 2008) trials found that clopidogrel has an advantage over aspirin in preventing recurrent cerebral infarction (an 8.7% advantage), but these results were all using western demographics, and head-to-head studies of aspirin and clopidogrel in eastern populations are lacking. It is still not clear how effective aspirin and clopidogrel are in these populations. Thus, further studies concerning the clinical risk of DCR are needed, as well as the monitoring of DCR occurrence, and the analysis of genetic-related risk factors for stroke recurrence throughout the course of treatment.

## Data Availability

The original contributions presented in the study are included in the article/Supplementary Material, further inquiries can be directed to the corresponding author.
